# ﻿Taxonomic studies of bluish *Mycena* (Mycenaceae, Agaricales) with two new species from northern China

**DOI:** 10.3897/mycokeys.90.78880

**Published:** 2022-06-17

**Authors:** Qin Na, Zewei Liu, Hui Zeng, Binrong Ke, Zhizhong Song, Xianhao Cheng, Yupeng Ge

**Affiliations:** 1 Shandong Key Laboratory of Edible Mushroom Technology, School of Agriculture, Ludong University, Yantai 264025, China Ludong University Yantai China; 2 Institute of Edible Mushroom, Fujian Academy of Agricultural Sciences, Fuzhou 350011, China Institute of Edible Mushroom, Fujian Academy of Agricultural Sciences Fuzhou China; 3 National and Local Joint Engineering Research Center for Breeding and Cultivation of Featured Edible Mushroom, Fuzhou 350011, China National and Local Joint Engineering Research Center for Breeding and Cultivation of Featured Edible Mushroom Fuzhou China

**Keywords:** Mycenoid fungi, phylogeny, taxonomy, two new taxa

## Abstract

Bluish *Mycena* are rare, but constitute a taxonomically complex group. A total of eight bluish species in four sections have previously been reported from North America, Europe, Oceania and Asia. Two species with a blue pileus, collected in China during our taxonomic study of *Mycena* s.l., are described here as new to science: *Mycenacaeruleogrisea***sp. nov.** and *M.caeruleomarginata***sp. nov.** Detailed descriptions, line drawings and a morphological comparison with closely-related species, especially herbarium specimens of *M.subcaerulea* from the USA, are provided. The results of Bayesian Inference and Maximum Likelihood phylogenetic analyses of a dataset of 96 nuclear rDNA ITS and 20 nLSU sequences of 43 *Mycena* species are also presented. The morphological data and the results of the phylogenetic analyses support the introduction of *M.caeruleogrisea* and *M.caeruleomarginata* as new species. A taxonomic key to bluish *Mycena* species of sections *Amictae*, *Cyanocephalae*, *Sacchariferae* and *Viscipelles* is provided.

## ﻿Introduction

*Mycena* (Pers.) Roussel, with almost 600 species distributed worldwide, is one of the largest genera in Agaricales (He et al. 2019). Maas Geesteranus (1980, 1992a, 1992b) proposed an infrageneric classification of *Mycena*, based on a combination of macroscopic and microscopic features. In this classification, the species are defined macroscopically based on basidiomata colour (pileus, stipe and lamellae face and edge). Within *Mycena*, species of sect. Adonideae (Fr.) Quél., now treated as *Atheniella* Redhead, Moncalvo, Vilgalys, Desjardin & B.A. Perry, sect. Aciculae Kühner ex Singer and sect. Oregonenses Maas Geest., are well characterised by their bright colours, such as pink, red, white or yellow (Maas Geesteranus 1980). Members of sect. Calodontes (Fr. ex Berk.) Quél. are prominently violet and dark colours can also be observed in sect. Rubromarginatae Singer ex Maas Geest. (Robich 2003, 2016; Aravindakshan and Manimohan 2015; Aronsen and Læssøe 2016). In addition, the microscopic characters are also considered to be very important in the infrageneric division of *Mycena*, containing basidiospores, cheilocystidia, pileipellis and stipitipellis (Maas Geesteranus 1992a, 1992b; Robich 2003, 2016; Aravindakshan and Manimohan 2015; Aronsen and Læssøe 2016). No current published framework exists for the genus as a whole, however and the morphologically based classification of Maas Geesteranus (1992a, 1992b) has not been fully tested and validated. Some recent studies indicate that several *Mycena* sections, for example, sects. *Amparoina* T. Bau & Q. Na, *Calodontes* (Fr. Ex Berk.) Quél and *Sacchariferae* Kühner ex Singer, are apparently monophyletic, whereas others are not (Harder et al. 2010; Na and Bau 2019b). Several taxa, traditionally assigned to *Mycena*, such as the *Atheniella* group, have been removed from the genus and others may need to be incorporated into genera, such as *Cruentomycena* R.H. Petersen, Kovalenko & O.V. Morozova, *Favolaschia* (Pat.) Pat., *Hemimycena* Singer, *Panellus* P. Karst., *Resinomycena* Redhead & Singer and *Roridomyces* Rexer (Redhead and Singer 1981; Rexer 1994; Antonín and Noordeloos 2004; Petersen et al. 2008; Redhead et al. 2012).

Eight bluish *Mycena* in four sections have been documented so far. Amongst these species, five have been reported from the Northern Hemisphere: *M.subcaerulea* Sacc. in North America, *M.amicta* (Fries) Quél. and *M.cyanorhiza* Quél. in Europe, *M.indigotica* Wei & Kirschner and *M.lazulina* Har. Takah., Taneyama and Terashima & Oba in Asia (Smith 1947; Maas Geesteranus 1980, 1992a, 1992b; Perry 2002; Robich 2003; Aronsen and Læssøe 2016; Terashima et al. 2016; Wei and Kirschner 2019; Perry et al. 2020). A bluish tint is often present on the pileus or stipe of these five species, four of them being classified into three sections: sect. Amictae Alexander H. Smith ex Maas Geesteranus,sect. Sacchariferae and sect. Viscipelles Kühner, but *M.indigotica* has tubes confused with members of *Favolaschia* (Pat.) Pat. and not assigned any section (Smith 1947; Maas Geesteranus 1980, 1992a, 1992b; Perry 2002; Robich 2003; Aronsen and Læssøe 2016; Terashima et al. 2016; Wei and Kirschner 2019; Perry et al. 2020). The three known bluish *Mycena* species from the Southern Hemisphere are *M.caesiocana* Singer, *M.cyanosyringea* Singer and *M.interrupta* (Berkeley) Sacc. (Singer 1969; Singer and Gomez 1982; Grgurinovic 2003). These species are distributed in Oceania and South America, Australia, Chile, Costa Rica, New Caledonia and New Zealand, where they usually grow on dead woods, decaying logs or tree stumps in deciduous forests of trees, such as *Eucalyptusrobusta* Smith and *Persealingue* (Ruiz & Pav.) Nees and develop basidiomata under high temperatures (Singer 1969; Singer and Gomez 1982; Grgurinovic 2003). The three allied species can be easily recognised: *M.caesiocana* and *M.cyanosyringea* are well characterised by the presence of a storm-grey pileus and extremely small basidiomata (pileus diameter and stipe length all less than 3 mm) and *M.interrupta* has a blue stipe base (Singer 1969; Singer and Gomez 1982; Grgurinovic 2003). In addition, *M.cyanocephala* Singer described from Chile, is considered to be synonymous with *M.interrupta* (Grgurinovic 2003). Although *M.cyanorhiza*, from the Northern Hemisphere, also has a blue stipe base similar to *M.interrupta*, but differs in pale brown to pale grey pileus and smaller basidiospores and cheilocystidia (Robich 2003; Aronsen and Læssøe 2016; Perry et al. 2020).

To date, fewer than 100 species of *Mycena* have been documented from China; amongst them, 14 new species have been described in recent years (He and Fang 1994; Guo et al. 1999; Shih et al. 2014; Li et al. 2015; Na and Bau 2018, 2019a, 2019b; Liu et al. 2021). During our investigations of mycenoid fungi in north-western China, we discovered two putative new taxa possessing a blue pileus with a greyish or brownish tint and a gelatinous pileipellis, clearly distinct from other species of *Mycena*, in the Liupan and Changbai Mountains. The results of our morphological observations and phylogenetic analyses support the introduction of these two new taxa.

## ﻿Materials and methods

### ﻿Morphology

Macromorphological observations were made on fresh specimens in the field and from photographs, with colour terms and notation following Kornerup and Wanscher (1978). Specimen pieces were mounted in 5% potassium hydroxide (KOH) and stained with Congo red when necessary. The prepared specimens were observed under a Lab A1 microscope (Carl Zeiss AG, Jena, Germany) and photographed and recorded using the supplied ZEN 2.3 (blue edition) software (Carl Zeiss AG). Melzer’s Reagent was used to test whether spores and tissues were amyloid and dextrinoid (Horak 2005). The dimensions of basidiospores were recorded according to Ge et al. (2021), Liu et al. (2021) and Na et al. (2021, 2022). The examined collections have been deposited in the Fungarium of the Fujian Academy of Agricultural Sciences (FFAAS), China. In the subsequent taxonomic description, author abbreviations follow Index Fungorum (http://www.indexfungorum.org).

### ﻿Phylogenetic analysis

Genomic DNAs of the putative new species were extracted from dried materials using a NuClean PlantGen DNA kit (Kangwei Century Biotechnology, Beijing, China). The internal transcribed spacer (ITS) region and the nuclear large subunit (nLSU) of nuclear ribosomal DNA were amplified using the PCR cycling protocol detailed in Ge et al. (2021) with primers ITS1/ITS4 and LR0R/LR7, respectively (White et al. 1990; Hopple and Vilgalys 1999). In addition, no sequence information has been published for *M.subcaerulea* and only a few ITS sequences of *M.cyanorhiza* and *M.amicta*, which were found to be phylogenetically closely related to the new species, are available in GenBank. For three *M.subcaerulea* specimens, we tried to obtain our target sequences by using next-generation sequencing (NGS) technology and whole-genome sequencing of the specimens was performed on the Illumina sequencing platform (HiSeq PE150) with standard procedures. The 150 bp paired-end libraries were prepared to generate approximately 3G raw data. ITS (GenBank accessions KT900146, NR_154169) and nLSU (GenBank accessions MK629349 and NG_070530) were randomly selected for using as custom seed and custom label databases according to the instructions (https://github.com/Kinggerm/GetOrganelle/wiki/FAQ: How to assemble custom loci?) of the software programme GetOrganelle (Jin et al. 2020). Finally, two ITS sequences (GenBank accessions OL711671 and OL711672) and three nLSU sequences (OL711666, OL711667 and OL711668) were captured from next-generation sequencing data of three specimens (TENN-F-051121, TENN-F-057919 and CUP-A-015335) of *M.subcaerulea* and used for subsequent analysis. Thirteen sequences (six ITS and seven nLSU) newly generated in this study were deposited in GenBank. Additionally, a total of 103 ITS and nLSU sequences (including *Xeromphalinacampanella* [Batsch] Kühner & Maire, which is often chosen as an outgroup for *Mycena*) were retrieved from GenBank for use in the phylogenetic analysis. Information on all analysed sequences (116) is given in Table 1. Generated sequences and those retrieved from GenBank were aligned and manually checked using BioEdit 7.0.4.1 and Clustal X 1.81 (Thompson et al. 1997; Hall 1999), with gaps in the alignment treated as missing data. The ITS and nLSU datasets were aligned separately. After estimating the optimal model of nucleotide evolution for the two partitions independently using Modeltest 3.7 (Posada and Crandall 1998), the two datasets were concatenated. The combined aligned dataset, which was deposited in TreeBase (submission ID 29069; study accession URL: http://purl.org/phylo/treebase/phylows/study/TB2:S29069), was subjected to Bayesian Inference (BI) and Maximum Likelihood (ML) phylogenetic analyses. The BI analysis was performed in MrBayes 3.2.6 (Ronquist and Huelsenbeck 2003). For the BI analysis, Markov Chain Monte Carlo chains were run for two million generations, with sampling carried out every 100^th^ generation until the critical value for the topological convergence diagnostic was less than 0.01 (Ronquist and Huelsenbeck 2003). The ML analysis, with a rapid bootstrapping algorithm involving 1,000 replicates, was performed in raxmlGUI 1.5b1 (Stamatakis et al. 2005).

**Table 1.** Specimens along with GenBank accession numbers used in the phylogenetic analysis. Sequences newly generated in this study are indicated in bold.

No.	Species	Voucher	Origin	ITS ID	LSU ID	References
1.	*Mycenaabramsii*	231a	Venice	JF908400	—	Unpublished
2.	*M.abramsii*	HMJAU 43282	China	MH396626	MK629348	Unpublished
3.	*M.abramsii*	HMJAU 43468	China	MH396627	—	Unpublished
4.	*M.abramsii*	KA12-0434	Korea	KR673481	—	Kim et al. (2015)
5.	*M.adscendens*	Aronsen120803	Norway	KT900140	—	Aronsen and Larsson (2015)
6.	*M.adscendens*	Orstadius329-05	Norway	KT900141	—	Aronsen and Larsson (2015)
7.	*M.adscendens*	Aronsen061119	Norway	KT900142	—	Aronsen and Larsson (2015)
8.	*M.adscendens*	Aronsen120826	Norway	KT900143	—	Aronsen and Larsson (2015)
9.	*M.albiceps*	MGW1504	USA	KY744173	MF797661	Unpublished
10.	*M.albiceps*	SAT1518708	USA	KY777372	MF797659	Unpublished
11.	*M.alnetorum*	CM14-RG2	USA	KU295552	—	Unpublished
12.	*M.amicta*	189f	Italy	JF908394	—	Osmundson et al. (2013)
13.	*M.amicta*	4745-HRL 1312	Canada	KJ705188	—	Unpublished
14.	*M.amicta*	CBS 352.50	France	MH856655	—	Vu et al. (2019)
15.	*M.amicta*	CBS 254.53	France	MH857183	—	Vu et al. (2019)
16.	*M.amicta*	CBS 257.53	France	MH857184	MH868722	Vu et al. (2019)
17.	*M.amicta*	H6036851	Finland	MW540687	—	Unpublished
18.	*M.arcangeliana*	252b	Italy	JF908401	—	Osmundson et al. (2013)
19.	*M.arcangeliana*	252f	Italy	JF908402	—	Osmundson et al. (2013)
20.	***M.caeruleogrisea***	**FFAAS 0001 Holotype**	**China**	**MW051896**	**OL711662**	**This study**
21.	***M.caeruleogrisea***	**FFAAS 0002**	**China**	**MW051897**	**OL711663**	**This study**
22.	***M.caeruleomarginata***	**FFAAS 0357 Holotype**	**China**	**OL711669**	**OL711664**	**This study**
23.	***M.caeruleomarginata***	**FFAAS 0358**	**China**	**OL711670**	**OL711665**	**This study**
24.	*M.chlorophos*	ACL257	Malaysia	KJ206983	—	Chew et al. (2015)
25.	*M.chlorophos*	ACL271	Malaysia	KJ206986	—	Chew et al. (2015)
26.	*M.cinerella*	Aronsen051014	Norway	KT900146	—	Aronsen and Larsson (2015)
27.	*M.cinerella*	173	Russia	MF926553	—	Malysheva et al. (2017)
28.	*M.citrinomarginata*	317h	Italy	JF908416	—	Osmundson et al. (2013)
29.	*M.citrinomarginata*	AD4TN	Tunisia	KU973883	—	Unpublished
30.	*M.clavicularis*	615i	Italy	JF908466	—	Osmundson et al. (2013)
31.	*M.clavicularis*	615b	Italy	JF908467	—	Osmundson et al. (2013)
32.	*M.cyanorhiza*	120b	Italy	JF908385	—	Osmundson et al. (2013)
33.	*M.deeptha*	DM334g	India	JX481737	—	Aravindakshan et al. (2012)
34.	*M.diosma*	KA13-1230	Korea	KR673698	—	Kim et al. (2015)
35.	*M.diosma*	320f	Italy	JF908417	—	Osmundson et al. (2013)
36.	*M.entolomoides*	HMJAU 43048	China	MG654736	—	Na and Bau (2018)
37.	*M.entolomoides*	HMJAU 43052	China	MG654737	MK722348	Na and Bau (2018)
38.	*M.entolomoides*	HMJAU 43126	China	MG654738	MK722349	Na and Bau (2018)
39.	*M.filopes*	3782	Canada	KJ705175	—	Unpublished
40.	*M.filopes*	KA12-1699	Korea	KR673631	—	Kim et al. (2015)
41.	*M.filopes*	287f	Italy	JF908410	—	Osmundson et al. (2013)
42.	*M.galericulata*	DM136-40516	USA	OM212953	—	Unpublished
43.	*M.galericulata*	LXL71	China	MZ669083	—	Unpublished
44.	*M.galericulata*	F26441	USA	MZ317346	—	Unpublished
45.	*M.galericulata*	EP.19-A1625	Greece	MT458520	—	Unpublished
46.	*M.galericulata*	50	Norway	MW576935	—	Unpublished
47.	*M.galericulata*	TFB14649	USA	MN088382	—	Unpublished
48.	*M.illuminans*	ACL161	Malaysia	KJ206975	—	Chew et al. (2015)
49.	*M.illuminans*	ACL175	Malaysia	KJ206976	—	Chew et al. (2015)
50.	*M.illuminans*	ACL212	Malaysia	KJ206980	—	Chew et al. (2015)
51.	*M.leaiana*	1028	Italy	JF908376	—	Osmundson et al. (2013)
52.	*M.leaiana*	CNH03 (TENN)	USA	MF686520	—	Unpublished
53.	*M.meliigens*	39	Italy	JF908423	—	Osmundson et al. (2013)
54.	*M.meliigena*	39d	Italy	JF908429	—	Osmundson et al. (2013)
55.	*M.metata*	313b	Italy	JF908412	—	Osmundson et al. (2013)
56.	*M.olivaceomarginata*	GG436-86	Svalbard	GU234119	—	Geml et al. (2015)
57.	*M.olivaceomarginata*	CBS 228.47	France	MH856228	MH867756	Vu et al. (2019)
58.	*M.olivaceomarginata*	CBS 229.47	France	MH856229	MH867757	Vu et al. (2019)
59.	*M.olivaceomarginata*	HK47-15	Norway	MT153141	—	Thoen et al. (2020)
60.	*M.pachyderma*	979a	Italy	JF908491	—	Osmundson et al. (2013)
61.	*M.pearsoniana*	FCME25817	USA	JN182198	—	Harder et al. (2012)
62.	*M.pearsoniana*	TENN61544	USA	JN182199	—	Harder et al. (2012)
63.	*M.pearsoniana*	TENN61384	USA	JN182200	—	Harder et al. (2012)
64.	*M.pelianthina*	CBH164	Denmark	FN394548	—	Unpublished
65.	*M.pelianthina*	108b	Italy	JF908379	—	Osmundson et al. (2013)
66.	*M.pelianthina*	108f	Italy	JF908380	—	Osmundson et al. (2013)
67.	*M.plumbea*	JN198391	China	JN198391	—	Wu et al. (2013)
68.	*M.plumbea*	420526MF0010	China	MG719769	—	Unpublished
69.	*M.polygramma*	439b	Italy	JF908433	—	Osmundson et al. (2013)
70.	*M.polygramma*	439f	Italy	JF908434	—	Osmundson et al. (2013)
71.	*M.pura*	TENN65043	USA	JN182202	—	Harder et al. (2012)
72.	*M.pura f. alba*	CBH410	USA	FN394595	—	Unpublished
73.	*M.purpureofusca*	SL09-06	Canada	HQ604766	—	Unpublished
74.	*M.purpureofusca*	G. Alfredsen	Norway	JQ358809	—	Unpublished
75.	*M.rosea*	938a	Italy	JF908488	—	Osmundson et al. (2013)
76.	*M.rosea*	Champ-21	Spain	KX449424	—	Pérez-Izquierdo et al. (2017)
77.	*M.rubromarginata*	407q	Italy	JF908430	—	Osmundson et al. (2013)
78.	*M.rubromarginata*	TL-12780	USA	KX513845	KX513849	Perry and Desjardin (2016)
79.	*M.seminau*	ACL136	Malaysia	KF537250	KJ206952	Chew et al. (2015)
80.	*M.seminau*	ACL308	Malaysia	KF537252	KJ206964	Chew et al. (2015)
81.	*M.seynesii*	71l	Italy	JF908469	—	Osmundson et al. (2013)
82.	*M.seynesii*	71h	Italy	JF908470	—	Osmundson et al. (2013)
83.	*M.silvae-nigrae*	515	Italy	JF908452	—	Osmundson et al. (2013)
84.	*M.silvae-nigrae*	CC 13-12	USA	KF359604	—	Baird et al. (2014)
85.	*M.stylobates*	455	Italy	JF908439	—	Osmundson et al. (2013)
86.	***M.subcaerul***ea	**TENN-F-051121**	**USA**	**OL711671**	**OL711666**	**This study**
87.	***M.subcaerulea***	**TENN-F-057919**	**USA**	**OL711672**	**OL711667**	**This study**
88.	***M.subcaerulea***	**CUP-A-015335**	**USA**	—	**OL711668**	**This study**
89.	*M.supina*	128a	Italy	JF908388	—	Osmundson et al. (2013)
90.	*M.tenax*	p187i	USA	EU669224	—	Unpublished
91.	*M.tenax*	OSC 113746	USA	EU846251	—	Unpublished
92.	*M.viridimarginata*	104h	Italy	JF908378	—	Osmundson et al. (2013)
93.	*M.vulgaris*	447h	Italy	JF908435	—	Osmundson et al. (2013)
94.	*M.vulgaris*	3781	Canada	KJ705177	—	Unpublished
95.	*M.zephirus*	KA13-1265	Korea	KR673722	—	Kim et al. (2015)
96.	*Xeromphalinacampanella*	TFB14487	USA	KP835678	KM011910	Aldrovandi et al. (2015)
97.	*X.campanella*	TFB7283A	USA	KM024575	KM024671	Aldrovandi et al. (2015)

## ﻿Results

### ﻿Phylogenetic analysis

**Figure 1.**
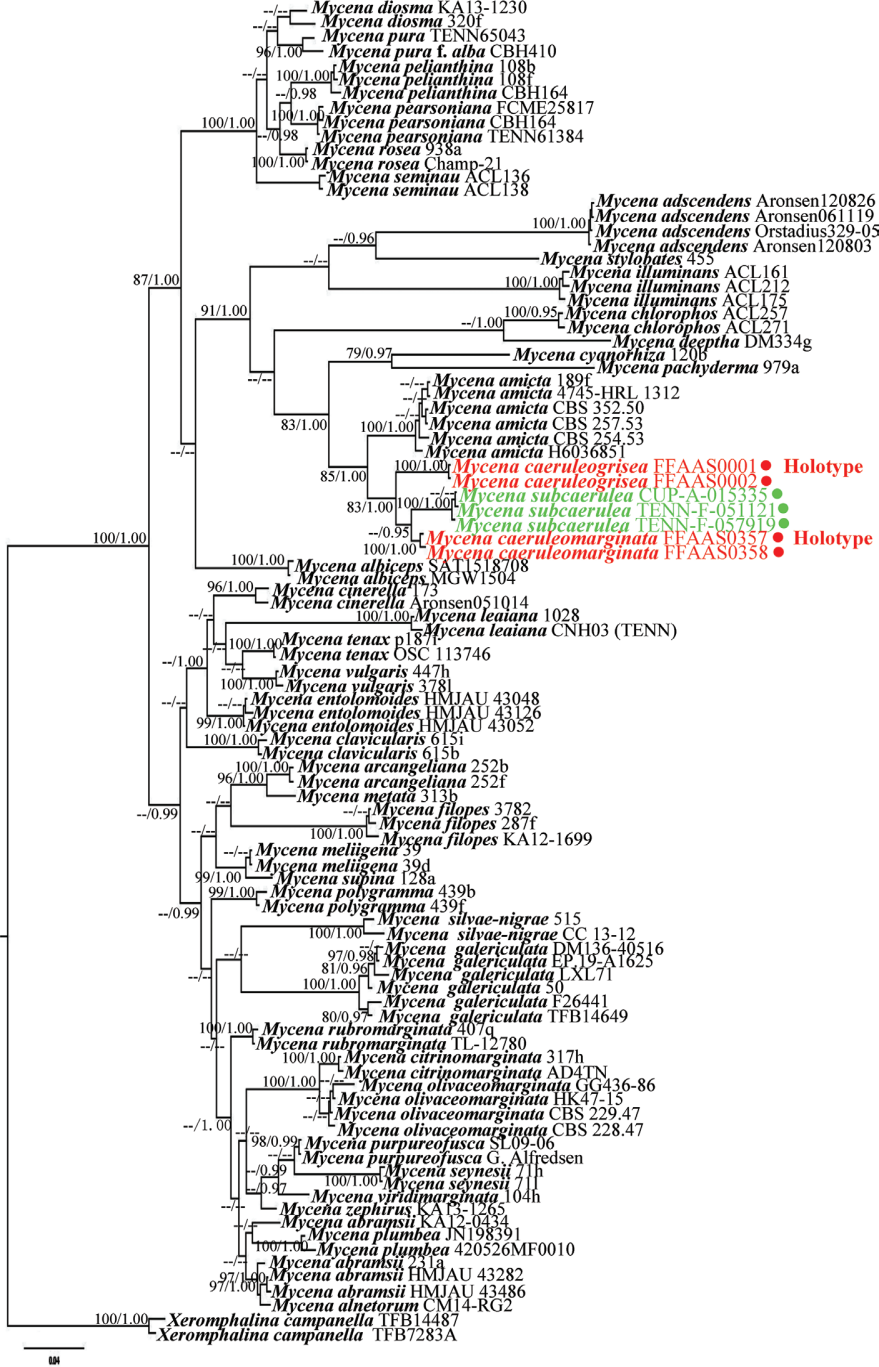
Phylogenetic tree inferred from partial ITS+nLSU sequence data by Bayesian inference and maximum likelihood. The tree is rooted with *Xeromphalinacampanella*. Maximum likelihood support values (BS) ≥ 75 and Bayesian posterior probabilities (BPP) ≥ 0.95 are indicated above or below branches (BS/BPP). Red dots indicate two new species, while green dots indicate *Mycenasubcaerulea* specimens from TENN and CUP.

BI and ML reconstructions, based on the optimal evolutionary model selected for the ITS and nLSU partitions (GTR + I + G), recovered similar topologies. The BI tree was selected as a representative phylogeny (Fig. 1).

In the tree shown in Fig. 1, which is based on 116 concatenated ITS+nLSU sequences of 43 *Mycena* species and the new taxa, the two samples of *M.caeruleogrisea* and the two samples of *M.caeruleomarginata* each form monophyletic lineages with high statistical support (*M.caeruleogrisea*, ML bootstrap support [BS] = 100, Bayesian posterior probability [BPP] = 1.00; *M.caeruleomarginata*, BS = 100, BPP = 1.00). According to the tree topology, *M.subcaerulea* is the species most closely related to *M.caeruleogrisea* and *M.caeruleomarginata*, consistent with morphology and clusters with the latter two species with high statistical support (BS = 100, BPP = 1.00). The *M.subcaerulea* clade comprises three samples: CUP-A-015335 (originally identified as *M.cyanothrix* G.F. Atk.), TENN-F-051121 and TENN-F-057919 (BS = 100, BPP = 1.00). By its morphological features and phylogenetic placement, sample CUP-A-015335 should be re-assigned to *M.subcaerulea*. The clade comprising *M.subcaerulea* and the two new taxa are sister to *M.amicta*, with the clade constituted by these four species in turn sister to *M.cyanorhiza*. Despite the close relationships, the two new species are strongly supported as distinct from *M.amicta* and *M.cyanorhiza* (Fig. 1).

It is noteworthy that the six samples of *M.amicta* from Europe and North America cluster together with strong support (BS = 100, BPP = 1.00), but the Canadian material (voucher no. 189f) seems to be closer to the Italian sample (voucher no. 4745-HRL 1312) than to the specimens from France and Finland. In addition, *M.pachyderma* Kühner, a non-bluish species in sect. Viscipelles, is a sister taxon (BS = 79, BPP = 0.97) to *M.cyanorhiza* in the same section.

## ﻿Taxonomy

In addition to morphological studies of the new taxa collected in China, morphological observations were made on 17 bluish specimens of *Mycena* loaned from fungal herbaria in the USA, namely, four specimens from the University of Tennessee (TENN) and 13 specimens from University of Cornell (CUP).

Our morphological observations using a light microscope confirmed the identity of 12 specimens as *M.subcaerulea*: TENN-F-014183, TENN-F-051121, TENN-F-052683, TENN-F-057919, CUP-A-002382, CUP-A-009686, CUP-A-014679, CUP-A-015138, CUP-A-015335, CUP-A-022677, CUP-A-023037 and CUP-A-023304. Another specimen, CUP-A-021234, previously identified as *M.iris*, was well characterised as *M.amicta*, based on its elongated ellipsoid basidiospores and clavate cheilocystidia with a rounded apex. As already noted by Smith (1947), the basidiomata of CUP-A-018443, CUP-A-022667, CUP-A-051322 and CUP-A-051323 were too small to be examined.

### ﻿ Mycena caeruleogrisea

Taxon classificationFungiAgaricalesMycenaceae

Q. Na, Y.P. Ge & H. Zeng sp. nov.

DB5C0797-7A85-59D6-B7C2-6C31018B57F8

 MB837656

Figs 2 , 3 , 4

#### Diagnosis.

This species is characterised by blue pileus, turning bluish-grey with age, pileus covered by a separable, gelatinous pellicle, stipe pruinose and with a blue base and stipe basal disc and acanathocysts of pileipellis absent. *Mycenasubcaerulea* differs from *M.caeruleogrisea* by a greenish-blue to greyish-brown pileus that turns yellow and remains blue at the centre and margin with age, a greenish-blue to brownish-blue stipe and smaller, globose to subglobose basidiospores.

#### Holotype.

China. Ningxia Hui Autonomous Region: Liangdianxia, Liupan Mountains National Forest Park, Jingyuan County, Guyuan City, 35°21'74"N, 106°18'37"E, 19 July 2020, Qin Na, Yupeng Ge, Hui Zeng, Junqing Yan and Zewei Liu, *FFAAS 0001* (collection number MY0164).

#### Etymology.

Refers to the pileus colour: blue when young, becoming bluish-grey with age.

#### Description.

**Figure 2.**
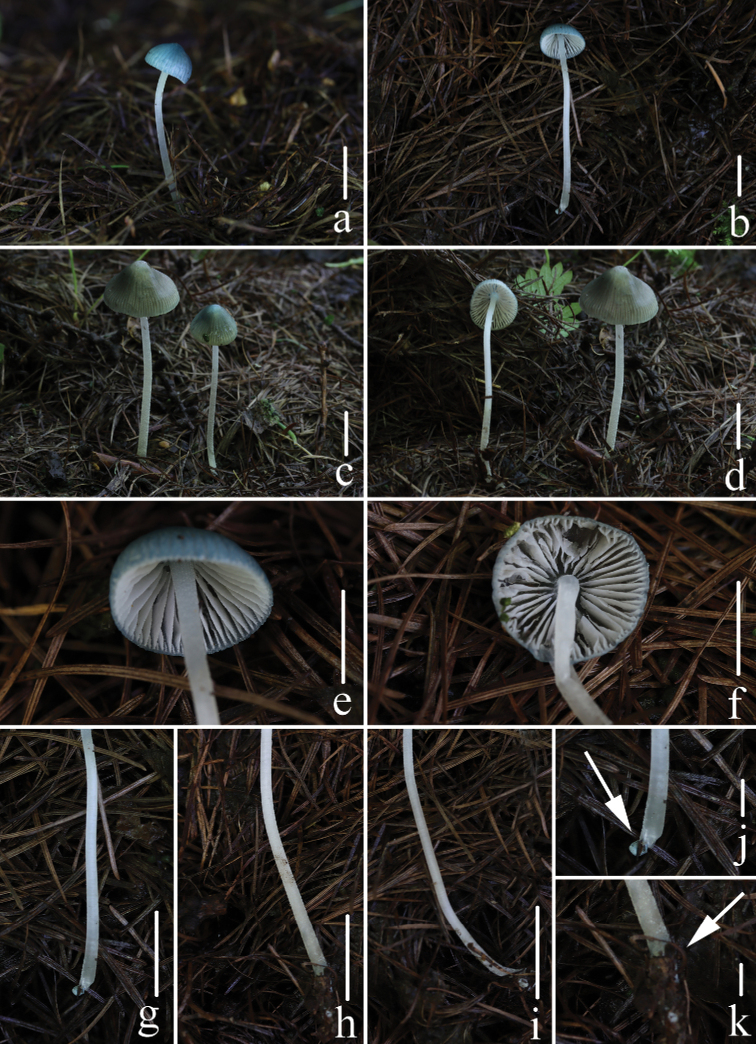
Fresh basidiomata of *Mycenacaeruleogrisea***a–d***M.caeruleogrisea* (*FFAAS 0001*, holotype) **e, f***M.caeruleogrisea* (*FFAAS 0002*) **g–i** entirely pruinose stipe **j, k** bluish base. Scale bars: 10 mm (**a–i**); 2 mm (**j–k**). Photographs by Yupeng Ge (**a, b**) and Qin Na (**c–k**).

Pileus 12–25 mm in diameter, hemispherical when young, conical, obtusely conical, campanulate with age, shallowly sulcate, translucently striate, almost smooth when young, becoming slightly brownish scaly at the centre, pruinose, with a glabrescent margin, dull blue (23D5) at the centre, margin pallescent to pastel blue (23A4), turning bluish-grey (23D2–23D3), a bit sticky, covered by a separable, gelatinous pellicle. Context white, thin, fragile. Lamellae 16–28 reaching the stem, adnate to slightly adnexed with a short tooth, narrowly spaced, white, with intervenose veins, edges concolorous with the face. Stipe 48–76 × 1.5–2.0 mm, equal or slightly broadened below, hollow, fragile, entirely pruinose (Fig. 2g–i), white, base greyish-blue (23B5) (Fig. 2j, k), covered with white fibrils, a basal disc absent. Odour and taste indistinctive.

Basidiospores [60/3/2] (8.8) 9.3–**10.4**–11.3 (11.8) × (5.5) 5.7–**6.5**–6.9 (7.3) μm [*Q* = 1.57–1.68, **Q** = ***1.60*** ± 0.072] [holotype [40/2/1] (9.1) 9.4–**10.3**–11.3 (11.6) × (5.6) 6.0–**6.5**–6.9 (7.2) μm, *Q* = 1.55–1.63, **Q** = ***1.59*** ± 0.049], ellipsoid, hyaline in 5% KOH, smooth, guttulate, thin-walled, amyloid. Basidia 22–29 × 7–9 μm, 4- or 2-spored, clavate. Cheilocystidia 40–62 × 4–6 μm, clustered, abundant, elongated clavate or cylindrical, apically broadly rounded, thin-walled, hyaline, forming a sterile lamellae edge. Pleurocystidia absent. Pileipellis an ixocutis with 1–4 μm wide hyphae, smooth or sparsely coated with simple cylindrical excrescences or inflated cells, 3–11 × 1–2 μm, embedded in gelatinous matter; acanathocysts absent. Hypodermium undifferentiated. Hyphae of the stipitipellis 3–8 μm in diameter, smooth, hyaline; caulocystidia 38–69 × 6–8 μm, long cylindrical, smooth, transparent. All tissues dextrinoid. Clamps present in all tissues.

**Figure 3.**
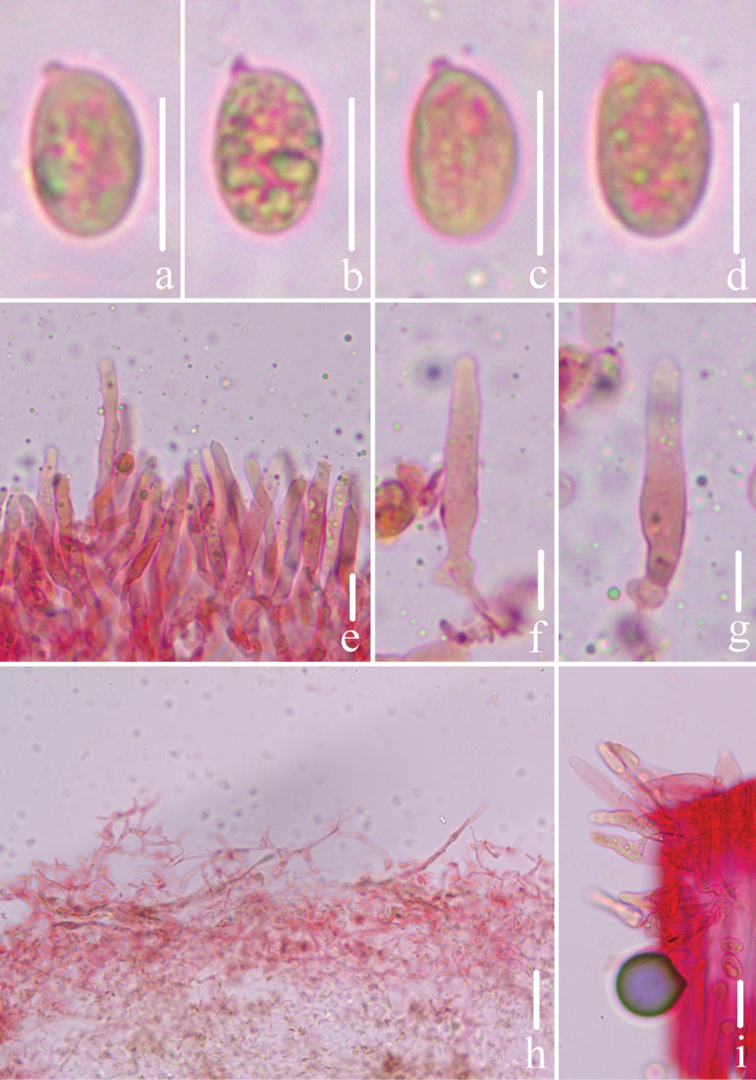
Microscopic features of *Mycenacaeruleogrisea* (*FFAAS 0001*, holotype) **a–d** basidiospores **e–g** cheilocystidia **h** pileipellis **i** stipitipellis and caulocystidia. Scale bars: 10 μm (**a–i**). Structures were stained with Congo Red medium before photographing.

#### Habit and habitat.

Scattered on humus and fallen leaves in mixed forests of *Acer*, *Populus*, *Pinus* and *Quercus*.

**Figure 4.**
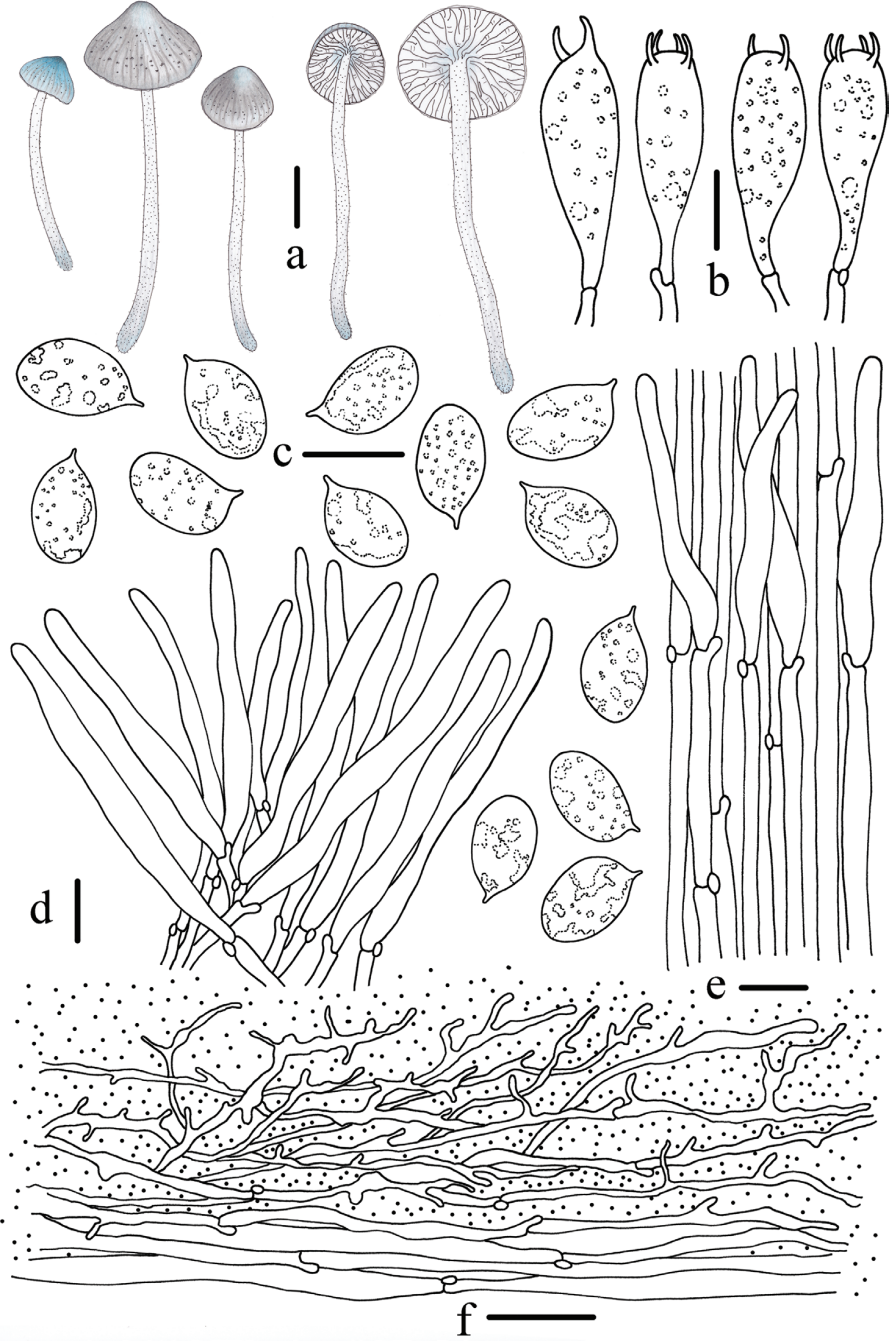
Morphological features of *Mycenacaeruleogrisea* (*FFAAS 0001*, holotype) **a** basidiomata **b** basidia **c** basidiospores **d** cheilocystidia **e** stipitipellis and caulocystidia **f** pileipellis. Scale bars: 10 mm (**a**); 10 μm (**b–f**). Drawings by Qin Na and Yupeng Ge.

#### Known distribution.

Ningxia Hui Autonomous Region, China.

#### Additional material examined.

Ningxia Hui Autonomous Region: Xiaonanchuan, Jingyuan County, Guyuan City, 20 July 2020, Qin Na, Yupeng Ge, Hui Zeng, Junqing Yan and Zewei Liu, *FFAAS 0002* (collection number MY0169).

#### Remarks.

**Figure 5.**
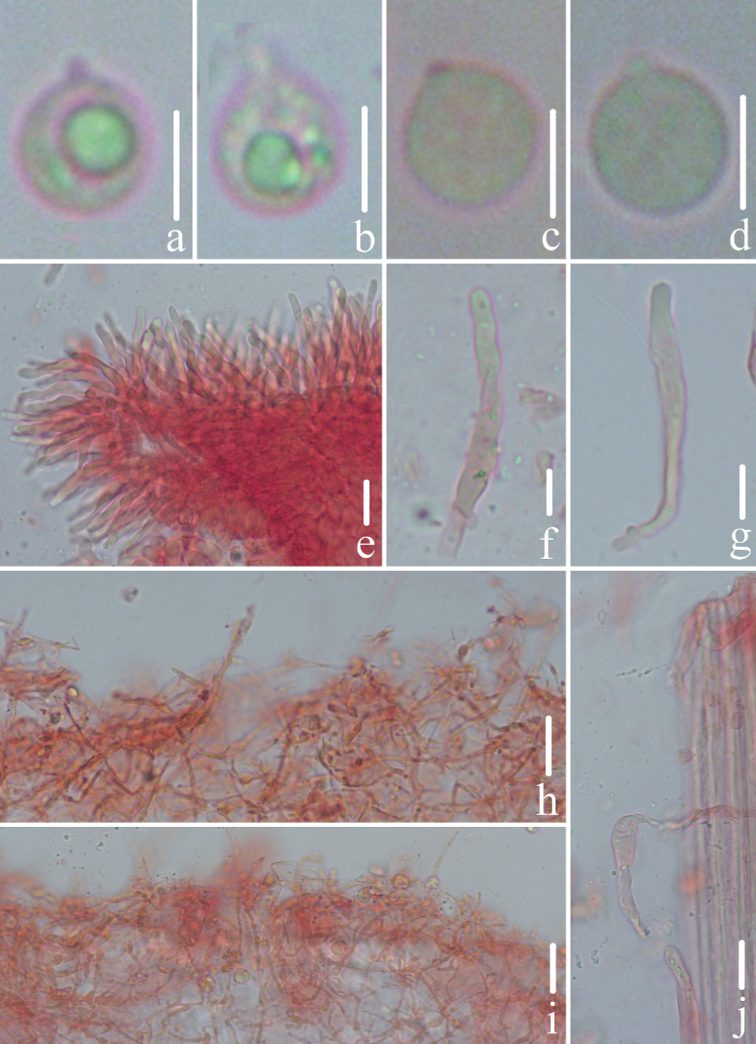
Microscopic features of *Mycenasubcaerulea***a, b** basidiospores (TENN-F-057919) **c** basidiospores (CUP-A-002382) **d** basidiospores (CUP-A-015138) **e–g** cheilocystidia (TENN-F-057919) **h, i** pileipellis (TENN-F-057919) **j** stipitipellis and caulocystidia (TENN-F-057919). Scale bars: 5 μm (**a–d)**; 10 μm (**e–j)**. Structures were stained with Congo Red medium before photographing.

**Figure 6.**
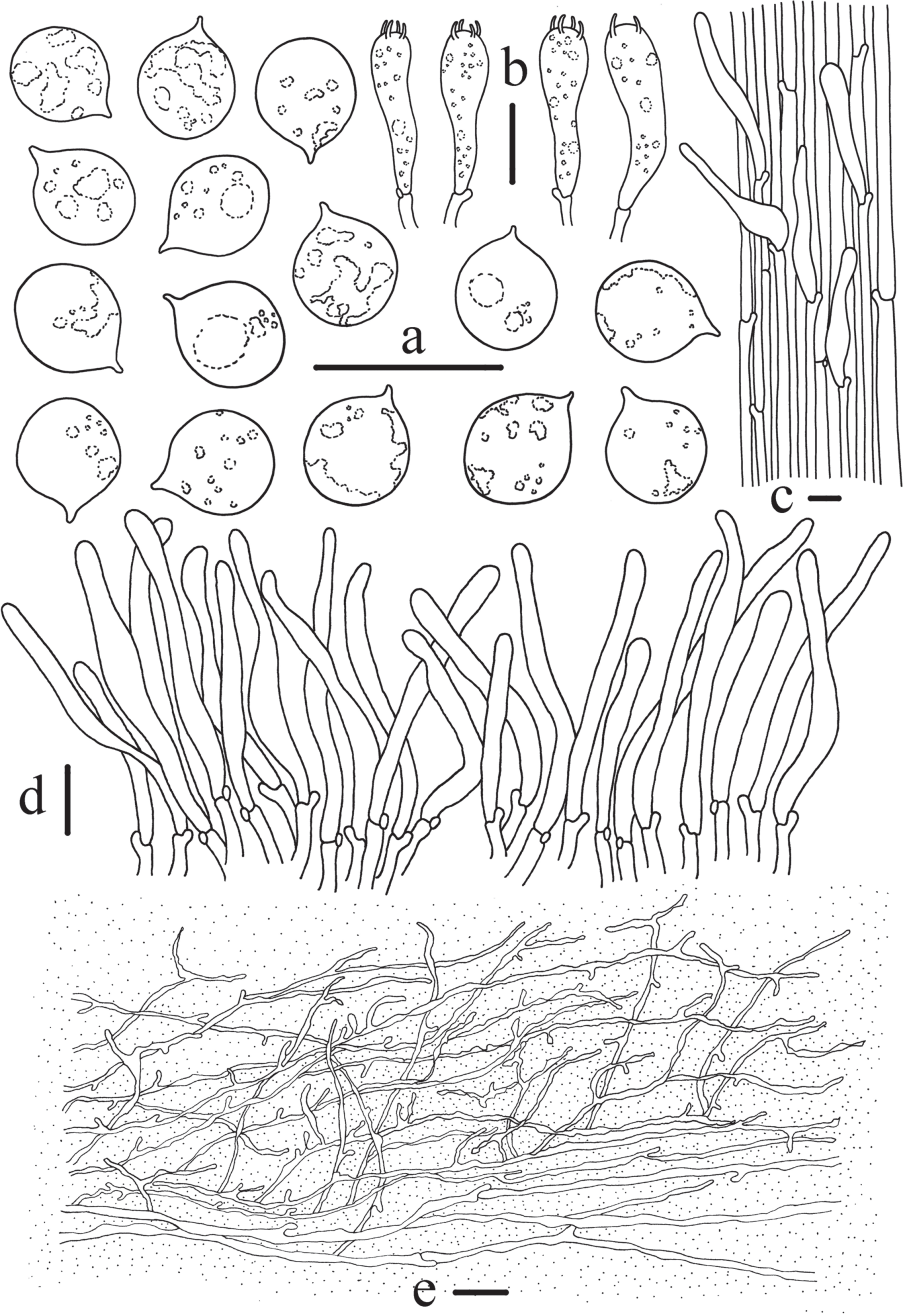
Morphological features of *Mycenasubcaerulea***a** basidiospores **b** basidia **c** stipitipellis and caulocystidia **d** cheilocystidia **e** pileipellis. Scale bars: 10 μm (**a–e**). Drawings by Qin Na and Yupeng Ge.

The original description of *M.subcaerulea* Sacc. was as follows: “*Pileo tenuissimo, campanulato v. convexo, striato, glabro, pallide cæruleo-viridi; stipite tenui, æquali, roseo-albo, subtiliter pruinoso; lamellis angustis, confertis, antice attenuatis, candidis; sporis subglobosis. 4 µ. d. Hab. In trunco fagineo in montibus Adirondack Amer. bor. – Cæspitosa, 5 cm. alta; pileus 8–13 mm. latus. Discus margine saturatius coloratus atque pileus cuticula secernibili obtectus.*” (Saccardo 1887). This North American species, which also has bluish basidiomata, is the taxon most closely resembling *M.caeruleogrisea* in both macro- and microscopic features; however, *M.subcaerulea* differs by a greenish-blue to greyish-brown pileus that turns yellow and remains blue at the centre and margin with age, a greenish-blue to brownish-blue stipe and smaller, globose to subglobose, basidiospores [6–8 × 6–7(8) µm] (Saccardo 1887; Smith 1947). In addition, *M.subcaerulea* was found solitary, scattered or gregarious on debris, decaying wood or bark around the bases of living trees, especially of oak, but also occurring quite frequently on decaying wood of basswood, elm, beech and other hardwoods (Smith 1947). The following microscopic characteristics of *M.subcaerulea* were also observed on the 11 CUP-A and TENN-F specimens in our study: basidiospores 5.6–8.3 × 5.3–7.9 µm, globose to subglobose; basidia 19–24 × 6–8 µm, clavate, 4-spored; cheilocystidia 36–55 × 3–6 µm; pileipellis hyphae 2–4 μm wide, coated with cylindrical excrescences or inflated cells, 1.1–14.9 × 0.7–1.4 μm, embedded in gelatinous matter; hyphae of the stipitipellis 4–10 μm in diameter; caulocystidia 42–70 × 4–10 μm, fusiform or cylindrical, smooth; clamps present (Figs 5, 6). In *M.cyanorhiza*, the base of the stipe can be strikingly sky blue, but it has a pale brown, grey to almost white pileus, a stipe base arising from a patch of fine fibrils, clavate to obpyriform cheilocystidia with finger-like excrescences and basidiospores that are elongated ellipsoid (*Q* = 1.6–2.2); these features all contrast with those of the new species (Aronsen and Læssøe 2016; Perry et al. 2020) (Table 2). In addition, *M.amicta* can be easily mistaken for *M.caeruleogrisea*, as it sometimes also has a bluish pileus when mature and similarly-shaped basidiospores, cheilocystidia and caulocystidia, but *M.amicta* can be distinguished from the latter species in having a pileus generally more brownish with a bluish tinge more or less present, an indistinct to raphanoid odour, a greyish-brown stipe that has a blue to blue-green base and is covered with a dense, fairly coarse, white pubescence and smaller cheilocystidia (16–45 × 3.5–7 µm); in addition, *M.amicta* is restricted to growth on wood and woody debris (Robich 2003; Aronsen and Læssøe 2016) (Table 2). *Mycenainterrupta*, which is well characterised by its acid blue to dull blue pileus and translucent stipe, is easily distinguished from *M.caeruleogrisea* by having smaller basidiomata, free lamellae, a white hirsute basal disc with blue margins on the stipe, broadly ellipsoid to subglobose spores and cheilocystidia covered with coarse excrescences (Grgurinovic 2003) (Table 2). *Mycenalazulina*, a new taxon reported from south-western Japan, possesses a blue stipe and cheilocystidia with numerous excrescences, which can be used to differentiate it from *M.caeruleogrisea* (Terashima et al. 2016). Another recently-described species of *Mycena* from Taiwan, *M.indigotica*, has blue basidiomata; however, the cap has tubes similar to *Boletus* and possesses globose basidiospores (Wei and Kirschner 2019).

**Table 2.** Morphological comparison of *Mycenacaeruleogrisea*, *M.caeruleomarginata*, and related species.

Taxa	*M.caeruleogrisea*	*M.caeruleomarginata*	*M.subcaerulea*	*M.amicta*	*M.cyanorhiza*	*M.interrupta*
**Pileus**	12–25 mm diam., hemispherical when young, conical, obtusely conical, campanulate with age, smooth when young, becoming slightly brownish scaly at the center, pruinose, acid blue to dull blue at the center and margin pallescent, turning bluish gray, covered by a separable, gelatinous pellicle.	3.5–13 mm in diam., parabolic, obtusely conical when young, hemispherical, campanulate with age, with an umbo at the center, shallowly sulcate, translucent-striate, smooth, gelatinous slightly, the margin infrequently out of flatness, brown to dark brown, becoming acid blue to dull blue towards the margin, with a greyish white margin, covered by a separable, sticky pellicle.	(3)5–15(25) mm broad, more or less ovoid with an appressed or slightly incurved margin, becoming obtusely conic to campanulate, surface lubricous subviscid, glabrous or appearing somewhat granulose near the margin, translucent-striate, pellicle tenacious and completely separable, pale blue or greenish blue, soon tinged with brown and assuming various degrees of bluish, greenish, or grayish brown with a pallid margin, often sordid yellowish in age, bluish tints often lingering on the margin.	5–15 mm wide, conical to campanulate, ± sulcate, translucent-striate, finely puberulous, covered with a separable gelatinous pellicle, pale grey-brown or pale sepia brown, sometimes with an olivaceous, greenish or bluish green shade, margin often bluish green, or more rarely dingy citrine to ochraceous yellow.	2–5(–10) mm wide, covered with a (separable), gelatinous pellicle, at first ± globose, then hemispherical to parabolic, becoming convex or somewhat depressed, but also with a small papilla, sulcate, translucent-striate, pruinose, glabrescent, somewhat lubricous, initially pale brown, then pale grey with darked centre, becoming almost white with age.	16 mm in diam., up to 4 mm high, at first subglobose to ovoid-conical, with age becoming convex to shallowly so, slightly depressed at apex, shiny, gelatinous, minutely radially rugulose, ± pruinose in places, at first dull blue at apex, below apex, becoming dull blue towards margin; margin decurved, entire, sulcate, striate, faintly translucent-striate.
**Context**	White, thin, fragile.	White, fragile, thin.	Thin, pallid, pliant.	–	–	Very thin to moderately thick at apex, translucent white or translucent greyish white.
**Lamellae**	16–28 reaching the stem, adnate to slightly adnexed with a short tooth, narrowly spaced, white, with intervenose veins.	14–25 reaching the stem, adnate to slightly adnexed with a short tooth, white, with unconspicuous intervenose veins, edges concolorous with the face.	Close to crowded, 18–25 reach the stipe, two or three tiers of lamellulae, ascending-adnate, sometimes narrowly adnate or practically free, narrow to moderately broad, white or tinged grayish, edges slightly fimbriate.	17–25 reaching the stem, ascending, adnexed, greyish to greyish brown; edge whitish, at times yellowish, greenish or bluish near the cap margin.	9–14 reaching the stem, ascending, adnexed to fairly broadly adnate or almost free, sometimes with a pseudocollarium, whitish or pale grey; edge whitish and separable as an elastic-tough thread.	Free from stipe or adnately attached to obvious circular descent of pileal flesh, moderately close to distant, five to seven per quadrant, subventricose, moderately broad to broad; edge marginate, blue; sides minutely pruinose, white; with one or two series of lamellulae.
**Stipe**	48–76 × 1.5–2.0 mm, equal or slightly broadened below, hollow, fragile, pruinose, white, base acid blue in the whole age, covered with white fibrils.	32–46 × 1.0–2.0 mm, equal, base sometimes slightly broaden, fragile, hollow, pruinose, puberulous entirely when young, becoming sparely especially in the middle part when old, yellowish brown to light brown, base with acid blue tinge, covered with a bit white fibrils.	3–8 cm long, 1–2 (2.5) mm. thick, equal, terete, flexuous or strict, tubular, cartilaginous, elastic, at first densely pruinose or minutely pubescent over all form a dense coating of caulocystidia, somewhat glabrescent, base mycelioid, the mycelium blue at first but soon fading to white, bluish to greenish blue above at first, soon fading to grayish or finally sordid brownish.	40–70 × 0.5–2 mm, cylindrical, entirely covered with a dense and fairly coarse, white pubescence, greyish brown, usually somewhat paler at the apex, occasionally with a slight lilaceous or violaceous tint; base at times somewhat rooting, concolorous or with some blue-green stains or entirely blue, even the substrate may be stained blue.	5–30 (–70) × 0.5–1 mm, cylindrical, entirely puberulous, glabrescent in the middle part, pale grey to hyaline-white; base hirsute, sky blue (also in the flesh), springing from a patch of fine, radiating, white fibrils.	Up to 22 mm long, cylindrical, moist to dry, often pruinose especially towards base, translucent white, attached to substratum via white pruinose disc borne on a flattened dull blue base.
**Odor & taste**	Indistinctive	Indistinctive	Mild	Indistinct to raphanoid.	Smell none or reported as faintly nitrous; taste not recorded.	Odour not distinctive.
**Spores**	(9.0) 9.3–11.6 (11.8) × (6.0) 6.2–7.3 (7.7) μm, Q = 1.5–1.7, ellipsoid, amyloid.	(6.2) 6.4–7.7 (7.9) × (4.4) 4.7–5.8 (6.0) μm, Q = 1.23–1.54, broadly ellipsoid to ellipsoid, amyloid.	6–8 × 6–7 (8) μm, globose or subglobose, amyloid.	7.5–10.7 × 4.5–6 μm, Q = 1.5–1.9, Qav ≈ 1.6, pip-shaped, amyloid.	6.5–9 × 4–5 μm, Q = 1.6–2.2, Qav ≈ 1.8, pip-shaped, amyloid.	(54/3), 8.4–11.6 (x̄ = 9.9, SD = ± 0.7) × 5.7–8.8 (x̄ = 7.0, SD = ± 0.6) μm, Q = 1.4, broadly ellipsoidal rarely subglobose, with prominent short, oblique apiculus, amyloid.
**Basidia**	22–29 × 7–9 μm, 4- or 2-spored.	26–35 × 6–12 μm, 4- or 2-spored.	4-spored	30–40 × 6–7 μm, clavate, 4-spored.	18–25 × 6.5–11 μm, clavate, 4-spored.	(27/2), 21.6–39.8 (x̄ = 29.0, SD = ± 5.2) × 8.3–16.0 (x̄ = 11.6, SD = ± 2.6) μm, 4-spored, rarely 2-spored, sterigmata to 8.8 μm long.
**Cheilocystidia**	40–62 × 4–6 μm, clustered, abundant, long clavate or cylindrical, apically broadly rounded, thin-walled, hyaline, forming a sterile lamellae edge.	32–48 × 4–6 μm, abundant, clustered, cylindrical or long clavate, apically broadly rounded, thin-walled, hyaline, forming a sterile lamellae edge.	Abundant, 32–60 × 5–8 μm, subfusoid with obtuse apices but becoming more or less cylindric, sometimes flexuous, smooth, hyaline.	16–45 × 3.5–7 μm, clavate, subfusiform or more often cylindrical.	9–20 × 5.5–7 μm, embedded in gelatinous matter, clavate to obpyriform, with few, simple to branched excrescences, 3–14 × 1–1.5 μm.	Abundant, (30/1), 16.8–44.8 (x̄ = 25.5, SD = ± 6.55) × 5.6–13.6 (x̄ = 8.4, SD = ± 1.8) μm, filamentous, cylindrical, clavate to ovoid, sometimes ventricose at base, with nodulose excrescences.
**Pleurocystidia**	Absent	Absent	Not differentiated	Absent	Absent	Absent
**Pileipellis**	Hyphae 1–4 μm wide, sparse, smooth or sparsely coated with simple, cylindrical excrescences or inflated cells, 3.1–11.2 × 0.8–1.7 μm, embedded in gelatinous matter.	Hyphae 2–4 μm wide, with simple, cylindrical excrescences, 2.0–6.4 × 0.6–1.8 μm, embedded in gelatinous matter.	A thick gelatinous pellicle (blue color located along the surface of the pellicle in incompletely gelatinized hyphae).	Hyphae 2–4.5 μm wide, branched, anastomosing, smooth with scattered, cylindrical excrescences, embedded in a layer of gelatinous matter.	Hyphae 1.5–3.5 μm wide, embedded in gelatinous matter, very branched, covered with scattered, simple to branched excrescences, protruding through the gelatinous layer.	Hyphae (28/1), 2.8–8.0 (x̄ = 5.4, SD = ± 1.4) μm in diam., nodulose-diverticulate with dense nodulose to cylindrical excrescences, gelatinized.
**Stipitipellis**	Hyphae 3–8 μm in diameter, smooth, hyaline.	Hyphae 3–6 μm in diameter, smooth, hyaline.	–	Hyphae 2–3.5 μm wide, smooth	Hyphae 1–3 μm wide, smooth.	Hyphae (26/1), 1.6–3.2 (x̄ = 2.4, SD = ± 0.4) μm in diam., not gelatinized.
**Caulocystidia**	38–69 × 6–8 μm, long cylindrical, smooth, transparent.	19–40 × 4–8 μm, smooth, transparent, two shapes: fusiform or cylindrical.	Covered with numerous cystidia, elongated and flexuous.	50–145 × 8–11.5 μm, fusiform to subcylindrical.	Up to 60 × 7 μm, simple to furcate or somewhat branched.	Often fasciculate, (25/1), 50.6–128.0 (x̄ = 75.0, SD = ± 19.8) × 5.0–8.8 (x̄ = 6.3, SD = ± 1.1) μm, filamentous to slightly ventricose especially towards base, rarely bifurcate.
**Clamps**	Present	Present	Present	Present	Present	Present
**Habitat**	Scattered, on humus and fallen leaves in *Acer*, *Populus*, *Pinus*, and *Quercus* mixed forests.	Scattered, on rotten wood in *Picea*, *Pinus*, *Quercus*, *Robinia*, and *Tilia* mixed forests.	Single, scattered or gregarious on debris, decaying wood, or on the bark around the bases of live trees of oak in particular, but also occurring quite frequently on decaying wood of basswood, elm, beech, and other hardwoods.	On wood and woody debris, mostly from conifers but also deciduous trees, also among leaves and needles.	On conifers (*Picea*, *Pinus* and *Larix*) bark and twigs, often on small bark fragments deep in grass.	Generally gregarious, often abundant, rarely solitary or scattered, on fallen decayed logs or stumps of *Eucalyptus*, *Nothofagus*, *Bedfordia*, *Pinus*, etc. forest.
**Distribution**	China	China	North America (Alabama, Carolina, New York, Tennessee, Pennsylvania, Michigan); Canada (Nova Scotia, Ontario, Manitoba)	Europe (Scandinavia, Netherlands, Italy)	Europe (UK, Denmark, Italy)	Australia and New Zealand
**Occurrence time**	Summer to autumn.	Late summer to early autumn.	Spring to fall, more abundant locally in the spring.	Late summer to late autumn, rarely in spring.	Summer to autumn.	March to July.
**References**	This study	This study	Saccardo 1887; Smith 1947	Robich 2003; Aronsen and Læssøe 2016	Robich 2003; Aronsen and Læssøe 2016; Perry 2020	Grgurinovic 2003

### ﻿ Mycena caeruleomarginata

Taxon classificationFungiAgaricalesMycenaceae

Q. Na & Y.P. Ge sp. nov.

093C3F71-C5A2-5D7B-B263-957A8A293E23

 MB842100

Figs 7 , 8 , 9

#### Diagnosis.

This species is characterised by dark brown pileus with a blue margin and the stipe densely pruinose, entirely covered with puberulous hairs and stipe basal disc and acanathocysts of pileipellis absent. *Mycenasubcaerulea* differs from *M.caeruleogrisea* in having a pileus that is distinctly greyish-brown with a blue centre and margin, turning yellow with age, a stipe tinged greenish-blue and globose to subglobose basidiospores.

#### Holotype.

China. Jilin Province: Chixi Protection Station, Erdaobaihe Town, Antu County, Yanbian Korean Autonomous Prefecture, 42°46'35"N, 128°15'04"E, 3 July 2021, Qin Na, Yupeng Ge, Binrong Ke and Chi Yang, *FFAAS 0357* (collection number MY0337).

#### Etymology.

Refers to the pileus, which is blue at the margin.

#### Description.

**Figure 7.**
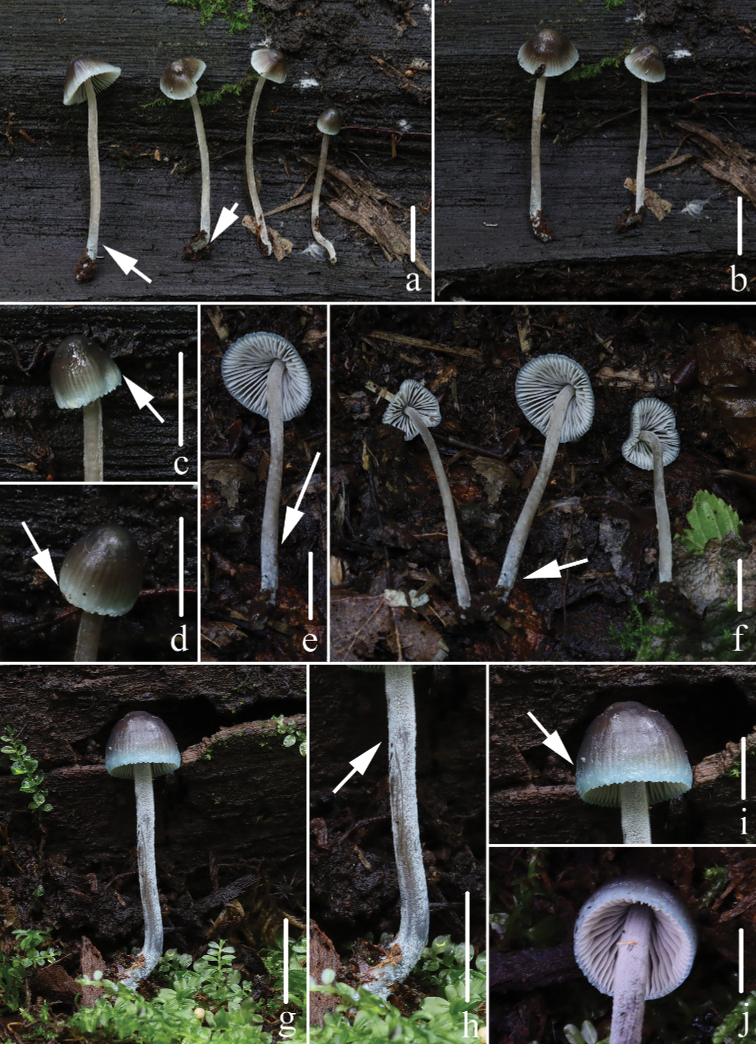
Fresh basidiomata of *Mycenacaeruleomarginata***a–f***M.caeruleomarginata* (*FFAAS 0357*, holotype) **g–j***M.caeruleomarginata* (*FFAAS 0358*) **a, f** stipe with a bluish base **c, d, i** pileus with blue margin **e, h** densely white, pruinose to pubescent stipe. Scale bars: 10 mm (**a, b, e, f, g, h**); 5 mm (**c, d**); 2 mm (**i, j**). Photographs by Qin Na (**a–f**) and Yupeng Ge (**g–j**).

Pileus 3.5–13 mm in diameter, parabolic, obtusely conical when young, hemispherical, campanulate with age, with an umbo at the centre, shallowly sulcate, translucently striate, smooth, slightly gelatinous, the margin infrequently out of flatness, dark brown (6F5–6F7), disc brown (6E6–6E7), becoming greyish-blue (23B5) to blue (23B7) towards the margin (Fig. 7c, d, i), margin grey (23B1) (Fig. 7c, d, i), covered by a separable, viscid pellicle. Context white, fragile, thin. Lamellae 14–25 reaching the stem, adnate to slightly adnexed with a short tooth, white, inconspicuously intervenose, edges concolorous with the face. Stipe 32–46 × 1.0–2.0 mm, equal, base sometimes slightly broadened, fragile, hollow, pruinose, entirely puberulous when young (Fig. 7h), becoming sparingly so, especially in the middle part, when old (Fig. 7e), greyish-brown (5E3) to brown (5E4), base with an greyish-blue (23B5) tinge (Fig. 7a, f), sparsely covered with white fibrils, a basal disc absent. Odour and taste indistinctive.

Basidiospores [60/3/2] (6.2) 6.4–**7.1**–7.7 (7.9) × (4.4) 4.7–**5.2**–5.8 (6.0) μm [*Q* = 1.23–1.54, **Q** = ***1.36*** ± 0.071] [holotype [40/2/1] (6.4) 6.6–**7.2**–7.7 (7.8) × (4.7) 4.9–**5.2**–5.3 (5.7) μm, *Q* = 1.26–1.53, **Q** = ***1.39*** ± 0.070], broadly ellipsoid to ellipsoid, hyaline in 5% KOH, guttulate, smooth, thin-walled, amyloid. Basidia 26–35 × 6–12 μm, 4- or 2-spored, clavate. Cheilocystidia 32–48 × 4–6 μm, abundant, clustered, cylindrical or elongated clavate, apically broadly rounded, thin-walled, hyaline, forming a sterile lamellae edge. Pleurocystidia absent. Pileipellis an ixocutis with 2–4 μm wide hyphae, simple, cylindrical excrescences, 2–6 × 1–2 μm, embedded in gelatinous matter; acanathocysts absent. Hypodermium undifferentiated. Hyphae of the stipitipellis 3–6 μm in diameter, smooth, hyaline; caulocystidia smooth, transparent, of two shapes: (1) fusiform or cylindrical, 19–40 × 4–8 μm; (2) extremely long cylindrical, sometimes with a narrow apex, 115–178 × 5–9 μm. All tissues dextrinoid. Clamps present in all tissues.

**Figure 8.**
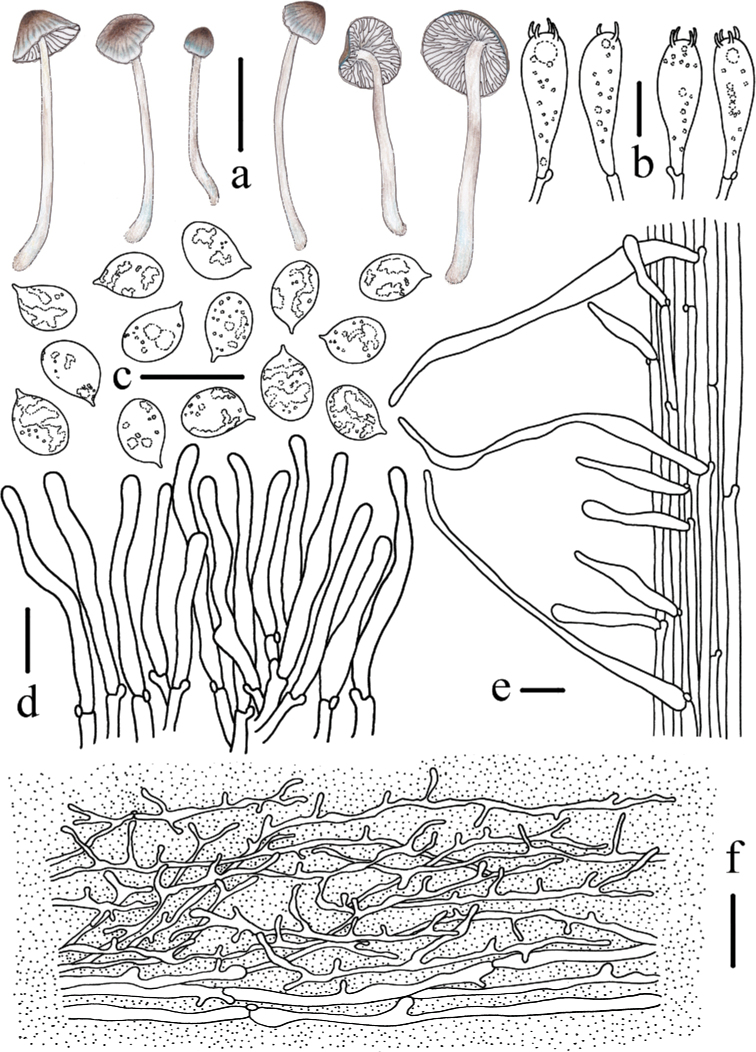
Morphological features of *Mycenacaeruleomarginata* (*FFAAS 0357*, holotype) **a** basidiomata **b** basidia **c** basidiospores **d** cheilocystidia **e** stipitipellis and caulocystidia **f** pileipellis. Scale bars: 10 mm (**a**); 10 μm (**b–f**). Drawings by Qin Na and Yupeng Ge.

#### Habit and habitat.

Scattered on rotten wood in *Picea*, *Pinus*, *Quercus*, *Robinia* and *Tilia* mixed forests.

**Figure 9.**
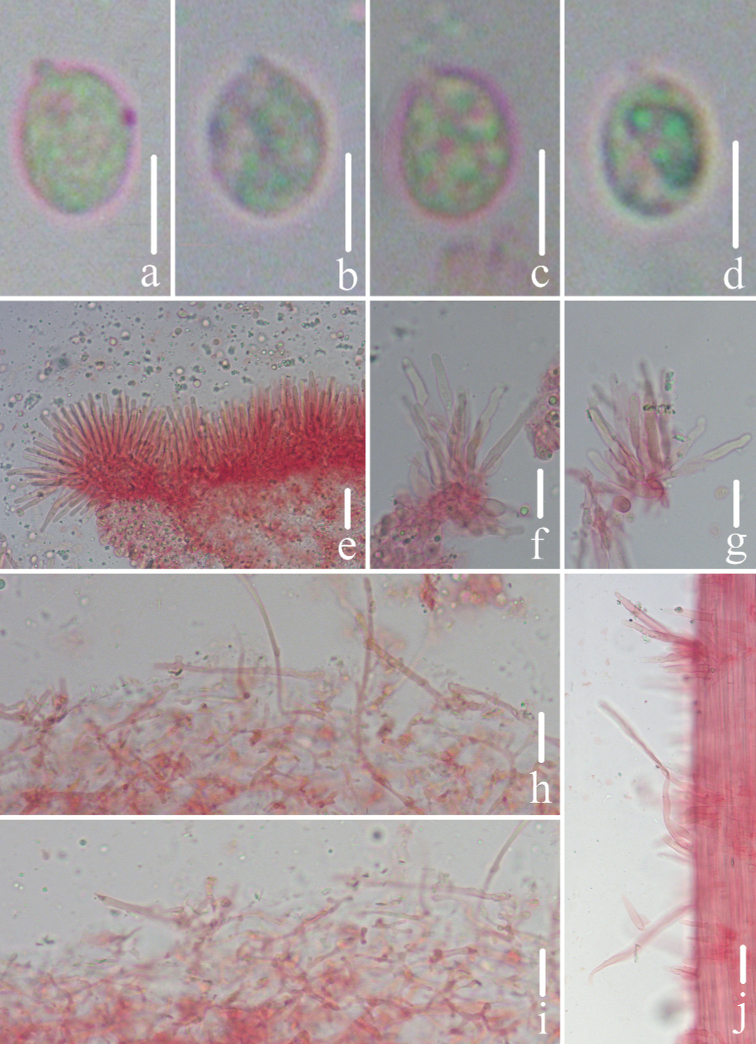
Microscopic features of *Mycenacaeruleomarginata* (*FFAAS 0357*, holotype) **a–d** basidiospores **e–g** cheilocystidia **h–j** pileipellis **j** stipitipellis and caulocystidia. Scale bars: 5 μm (**a–d)**; 10 μm (**e–j**). Structures were stained with Congo Red medium before photographing.

#### Known distribution.

Jilin Province, China.

#### Additional material examined.

Jilin Province: Hancongling, Erdaobaihe Town, Antu County, Yanbian Korean Autonomous Prefecture, 42°46'36"N, 128°15'04"E, 4 July 2021, Qin Na, Yupeng Ge, Binrong Ke and Chi Yang, *FFAAS 0358* (collection number MY0343).

#### Remarks.

The diagnostic features of *M.caeruleomarginata* can be used to distinguish this new taxon from the closely-related bluish species *M.subcaerulea*, *M.cyanorhiza*, *M.amicta* and *M.interrupta* (Table 2). *Mycenasubcaerulea*, the species most similar to *M.caeruleomarginata*, differs in having a pileus that is distinctly greyish-brown with a blue centre and margin, turning yellow with age, a stipe tinged greenish-blue and globose to subglobose basidiospores (*Q* = 1.01–1.14) according to the original description and our observations (Saccardo 1887; Smith 1947) (Figs 5, 6; Table 2). Similar to *M.caeruleomarginata*, *M.cyanorhiza* has an entirely puberulous, pruinose stipe with a sky blue base and possesses a gelatinous pileus; however, the pileus of *M.cyanorhiza* is pale brown, grey to almost white, without a bluish tinge and this species has elongated ellipsoid basidiospores (*Q* > 1.6) and lacks smooth cheilocystidia and caulocystidia (Aronsen and Læssøe 2016; Perry et al. 2020). In addition, *M.amicta* resembles *M.caeruleomarginata* in its bluish pileus, pruinose stipe and pileipellis embedded in a layer of gelatinous matter, but the former differs in having a pale grey-brown pileus that is sometimes ochraceous yellow and greenish when young and bluish when old, a raphanoid odour and elongated ellipsoid basidiospores (7.5–10.7 × 4.5–6.0 µm) (Robich 2003; Aronsen and Læssøe 2016). The Southern Hemisphere species *M.interrupta* is well characterised by its blue pileus at maturity, a translucent stipe with a basal disc and cheilocystidia with excrescences (Grgurinovic 2003). Furthermore, two new species with bluish basidiomata reported from East Asia, *M.lazulina* and *M.indigotica*, can be easily distinguished from the new species in their whitish pileus or tubes similar to *Boletus*; *M.lazulina* having cheilocystidia with numerous excrescences and *M.indigotica* possesses globose basidiospores (Terashima et al. 2016; Wei and Kirschner 2019). *Mycenacaeruleogrisea* and *M.caeruleomarginata* share the same bluish pileus and stipe base, smooth and cylindrical cheilocystidia and pileipellis embedded in a layer of gelatinous matter. *Mycenacaeruleomarginata* can be readily distinguished, however, based on the dark brown colour of the pileus with a blue margin, yellowish-brown to light brown stipe, broadly ellipsoid to ellipsoid spores and caulocystidia of two shapes.

### ﻿Key to seven bluish *Mycena* species of sections *Amictae*, *Cyanocephalae*, *Sacchariferae*, and *Viscipelles*

**Table:** 

1	Cheilocystidia non-smooth	**2**
–	Cheilocystidia smooth (sect. Amictae)	**4**
2	Acanthocysts present (sect. Sacchariferae)	***M.lazulina***
–	Acanthocysts absent	**3**
3	Stipe with basal disc (sect. Cyanocephalae)	***M.interrupta***
–	Stipe without basal disc (sect. Viscipelles)	***M.cyanorhiza***
4	Basidiospores subglobose	***M.subcaerulea***
–	Basidiospores broadly ellipsoid to ellipsoid	**5**
5	Caulocystidia of two types: (1) fusiform or cylindrical, 19–40 × 4–8 μm; (2) extremely long, cylindrical (length > 100 μm)	***M.caeruleomarginata***
–	Caulocystidia of one type, fusiform, subcylindrical to cylindrical (length < 100 μm)	**6**
6	Pileus pale grey-brown or pale sepia brown, sometimes with an olivaceous, greenish or bluish-green shade; margin often bluish-green or rarely dingy citrine to ochraceous yellow	***M.amicta***
–	Pileus sky blue, greyish-blue with age; margin blue when young, turning bluish-grey when old	***M.caeruleogrisea***

## ﻿Discussion

With their blue pileus and gelatinous pileipellis, the new taxa *M.caeruleogrisea* and *M.caeruleomarginata* are unique in China. Similar species described from North America and Europe, namely, *M.subcaerulea*, *M.cyanorhiza* and *M.amicta*, have bluish basidiomata as well, but with age, these species often change colours—to green, brown or yellow and the sizes and shapes of their basidiospores and cheilocystidia are also different (Saccardo 1887; Smith 1947; Maas Geesteranus 1980, 1992a, 1992b; Grgurinovic 2003; Robich 2003; Aronsen and Læssøe 2016) (Table 2). *Mycenainterrupta*, described from the Southern Hemisphere, can be distinguished from the two newly-described species, based on both habitat and morphology (Grgurinovic 2003). *Mycenalazulina* (sect. Sacchariferae), which has a white pileus, blue stipe base, acanthocysts and a non-gelatinised pileipellis, seems to be the most distinct bluish species and is not included in Table 2 (Terashima et al. 2016). According to taxonomic research based on morphology and phylogeny, our newly-described species are more similar to *M.subcaerulea* and *M.amicta* and should, thus, be classified into sect. Amictae.

Although pileus colour has been used as a basis for sectional division in *Mycena*, this character does not seem to be satisfactory for species identification, especially within the same section (Smith 1947; Maas Geesteranus 1980, 1992a, 1992b; Grgurinovic 2003; Robich 2003; Aronsen and Læssøe 2016). In sect. Viscipelles, for example, *M.cyanorhiza* can be distinctly characterised by the presence of a sky blue stipe, but *M.ulmi* B.A. Perry & H.W. Keller, *M.pachyderma* and *M.pseudocyanorrhiza* Robich do not exhibit any bluish tint (Robich 2003; Aronsen and Læssøe 2016; Perry et al. 2020). A combination of macroscopic and microscopic features, such as the colour of basidiomata and the shapes and sizes of spores, cheilocystidia, pileipellis, caulocystidia and dextrinoid tissues, is, thus, generally regarded as more important for the identification of *Mycena* taxa.

## Supplementary Material

XML Treatment for Mycena caeruleogrisea

XML Treatment for Mycena caeruleomarginata
